# In-Situ Controllable Fabrication and Performance of Laser-Induced Graphene Flexible Sensors for Human Motion Monitoring

**DOI:** 10.3390/s26216886

**Published:** 2026-10-30

**Authors:** Jun Zhang, Ping-An Yang, Fang He, Haochuan Wan, Yi Lu

**Affiliations:** 1 School of Robot Engineering, Yangtze Normal University, Chongqing 408100, China; 20140016@yznu.edu.cn (J.Z.); 19970012@yznu.edu.cn (F.H.); 20180002@yznu.edu.cn (H.W.); 2 School of Automation, Chongqing University of Posts and Telecommunications, Chongqing 400065, China; yangpa@cqupt.edu.cn; 3 School of Integrated Circuits, Chongqing University of Posts and Telecommunications, Chongqing 400065, China

**Keywords:** flexible strain sensors, laser-induced graphene, stability, strain range, human motion monitoring

## Abstract

Graphene-based resistive sensors exhibit excellent sensing performance, but face challenges in terms of uniformity, stability, and large-scale fabrication. To address these issues, in this study, we blade-coated a PI/PDMS precursor on the fabric surface and constructed graphene via in situ laser induction, thereby effectively improving the preparation efficiency and stability of graphene. Meanwhile, the influence of the PI-to-PDMS ratio on the performance of the laser-induced graphene resistive sensor was investigated. It was found that at a PI-to-PDMS ratio of 1:3, the formed graphene showed the fewest defects and the highest graphene proportion, which originated from its good fluidity, compatibility, and crosslinking density. Experimental results demonstrate that the sensor exhibits favorable sensing performance, with a linearity of 0.99 and a sensitivity of 8.64 within the 10–20% strain range of Sample 2, along with repeatable stability over 2000 cycles, meeting the requirements of skin-friendly contact and highly stable motion monitoring for human joints. This study provides a reference for the efficient and rapid construction of graphene-based resistive sensors and shows considerable application potential.

## 1. Introduction

With the advancement of new-generation information technology and the intensification of global aging, societal demands for medical monitoring [1,2] and motion rehabilitation [3,4] are becoming increasingly urgent. Owing to their low cost, lightweight design, and excellent integration features, flexible wearable devices [5] are being widely applied in health monitoring [6], human–computer interaction [7], and biomimetic robotics [8] through skin-attached or textile-integrated modalities. Among the current mainstream types of flexible sensors, such as resistive [9], capacitive [10,11], piezoelectric [12,13], and triboelectric [14,15], resistive sensors have emerged as a research focus in the fields of wearable devices [16,17] and electronic skin due to their notable advantages, including high sensitivity, fast response, wide linear range, simple fabrication process, and low cost, demonstrating significant application potential.

For resistive flexible sensors, structural design and the selection of sensing materials are key factors determining their performance. Commonly used sensing materials include metal-based nanomaterials [18,19] (such as silver nanowires, iron nanowires, MXene), carbon-based nanomaterials [20] (such as MWCNT, graphene), and conductive polymers [21] (such as PPY, PEDOT:PSS), among others. Among these materials, carbon nanocomposites have attracted extensive attention due to their excellent electrochemical and mechanical properties. In particular, graphene, with its unique mechanical strength and outstanding electrical and thermal properties, is widely used as the conductive functional layer for constructing flexible sensors [22,23]. Xu et al. [24] prepared a nanostructures@graphene foam strain sensor with a satisfactory sensitivity (GF) of 227 and a wide sensing range of up to 90%. However, traditional graphene fillers often suffer from poor dispersion and typically require high filling content to form a well-established conductive network. This not only increases fabrication complexity but may also compromise the inherent flexibility and mechanical properties of the composite material [25]. In addition, the compatibility between the conductive layer and the flexible substrate is also a critical factor affecting sensor longevity. Most existing preparation methods rely solely on simple surface physical adsorption to attach conductive materials onto the substrate. Such weak interactions result in insufficient interfacial bonding, making the conductive layer prone to peeling under long-term cyclic use or external friction, which severely degrades the stability of the sensor [26].

In response to this challenge, laser-induced graphene (LIG) technology offers an ideal solution [27]. As an efficient and economical micro-nanofabrication method, LIG technology achieves in situ graphitization of carbon-based precursors through laser direct writing, thereby constructing a robust integrated interface between the conductive layer and the substrate, significantly enhancing the overall stability of the device. However, the effectiveness of this process highly depends on the intrinsic properties of the precursor material. Although polydimethylsiloxane (PDMS) is the preferred substrate in flexible electronics due to its excellent rheological properties and high elasticity, its low carbon content limits the generation of high-quality graphene, resulting in insufficient conductivity of the sensor. Conversely, polyimide (PI) possesses high carbonization efficiency and thermal stability, but its inherent brittleness (ultimate elongation < 5%) severely restricts its application in high-strain scenarios. In light of this, constructing a PI/PDMS composite system has emerged as a key strategy to overcome the limitations of single-material performance. This approach utilizes the PDMS matrix to impart outstanding stretchability to the film, while the PI component provides an ample carbon source, thereby achieving an optimal balance between mechanical flexibility and electrical performance. This work aims to design a flexible strain sensor suitable for human joint motion monitoring through in situ construction of laser-induced graphene (LIG).

In this study, we fabricated a flexible strain sensor based on LIG using a PI/PDMS composite substrate. The research began by blending PDMS with PI to create a composite film that combines high flexibility with high carbonization efficiency, followed by the preparation of a conductive functional layer via laser direct writing. To balance the mechanical properties and electrical response of the sensor, the formulation ratio of the substrate was optimized, and the sensitivity, tensile limit, and cycling stability of the device under different carbon contents and surface morphologies were systematically investigated. Experimental results demonstrate that the sensor exhibits excellent overall performance. By fabricating it into an integrated sensing glove, finger joint movements were successfully captured, confirming the applicability of this strategy in the field of high-performance flexible wearable electronics.

## 2. Materials and Methods

### 2.1. Materials

The PDMS elastomer kit (Sylgard 184) was purchased from Dow Corning (Midland, MI, USA). Thermally conductive polyimide powder (PI) with an average particle size of approximately 16 μm (870 mesh) was obtained from Dongguan Zhangyang Polymer Materials Co., Ltd. (Dongguan, China). The thermally conductive silver paste (0.3 mL, JY59) was sourced from Shanghai Julong Electronics Technology Co., Ltd. (Shanghai, China). A thin nylon–spandex blend fabric was used as the textile substrate. A wire-wound applicator with a total length of 240 mm and a coating thickness of 10 μm was employed. The sensor mold, designed in a rectangular shape with a width of 40 mm and a depth of 1 mm, was fabricated using resin-based hydrogel material via 3D printing.

### 2.2. Sensor Preparation

**Figure 1:**
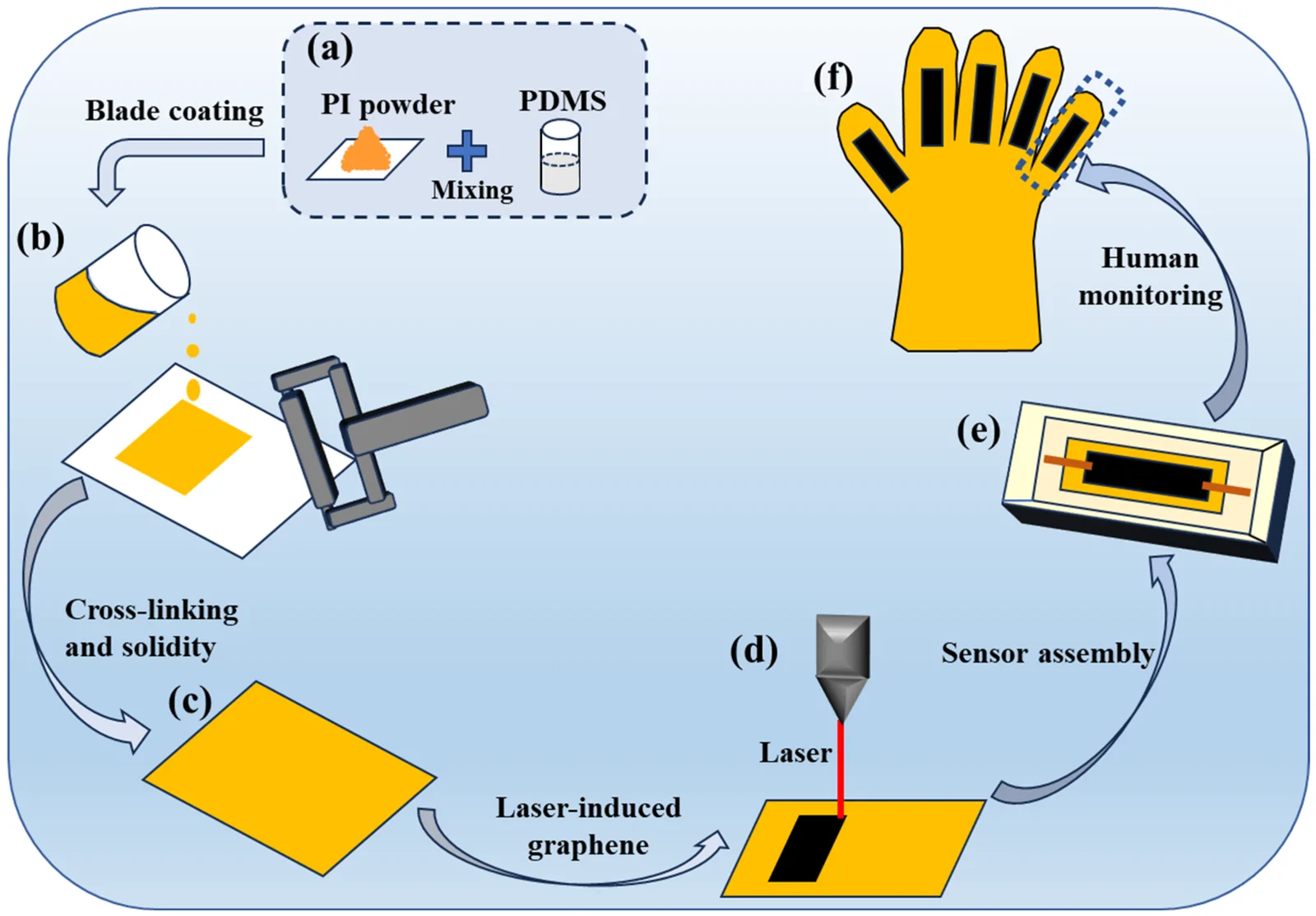
Schematic illustration of the fabrication process: (**a**) PI/PDMS mixture; (**b**) blade coating; (**c**) curing and drying; (**d**) laser-induced graphene; (**e**) sensor encapsulation; and (**f**) human-motion monitoring.

The fabrication process of the flexible sensor based on laser-induced in situ construction of graphene is illustrated in Figure 1. First, the PDMS solution was prepared by mixing 10 g of component A and 1 g of curing agent component B at a ratio of 10:1 in a beaker, followed by stirring evenly with a glass rod for 3 min to ensure thorough blending. Subsequently, PI powder was added to the PDMS at PI:PDMS mass ratios of 1:2, 1:3, and 1:4, with the amounts being 5.50 g, 3.67 g, and 2.75 g, respectively, and the mixtures were stirred until completely and uniformly dispersed. The mixture was placed in a vacuum chamber for degassing and left to stand until no bubbles remained in the solution. Then 10 mL of the prepared mixed solution (Figure 1a) was poured onto a prepared fabric with an area of 0.1 m^2^, which was fixed at all four ends onto a glass plate. This setup ensured the fabric remained level without tilting and facilitated uniform coating of the mixture. A blade was then used to evenly spread and impregnate the mixture onto the fabric, maintaining a consistent angle and pressure. The coated fabric was placed in a 60 °C drying oven for 4 h, resulting in the dried fabric impregnated with the PI/PDMS mixture as shown in Figure 1c. Subsequently, a commercial CO_2_ laser cutter (Model E5030, purchased from Golden Leap Laser Science and Technology Co., Ltd., Dongguan, China) with a wavelength of 10.6 µm was used for laser direct writing on the fabric. During laser direct writing, the laser power was set to approximately 3.9 W, the scanning speed to 40 mm/s, the beam size to about 150 µm, and the pulse width to about 50 µs. The resulting product, as shown in Figure 1d, consisted of a fabric base with graphene formed on top. Copper electrodes were attached to both ends of the sample using thermally conductive silver paste, and the assembly was placed in a 60 °C drying oven for 30 min. After removal, it was placed in a mold, filled with PDMS, and then cured in an 80 °C drying oven for 3 h to obtain the encapsulated graphene sensor, which was ready for human motion monitoring tests, as shown in Figure 1f.

To explore the optimal ratio of PI to PDMS and verify the versatility of different proportions, this study considered that a high PI content would reduce the fabric’s flexibility, while a low PI content would affect graphene formation. Therefore, three ratios—PI:PDMS of 1:2, 1:3, and 1:4—were selected to prepare three types of samples, hereafter referred to as Sample 1, Sample 2, and Sample 3, respectively.

### 2.3. Characterization and Analysis

The microscopic morphology of the fabric surface after laser induction was observed using a field-emission scanning electron microscope (SEM, Thermo Fisher Scientific Apreo2C, Waltham, MA, USA). Defects, layer count, and other characteristics of the graphene were analyzed by confocal Raman spectroscopy (Raman, Horiba Scientific Lab RAM HR Evolution, Longjumeau, France) with an excitation wavelength of 325 nm and a wavenumber range of 50–4000 cm^−1^. The surface chemical composition was determined using energy-dispersive spectroscopy (EDS, Oxford Ultim Max65, Abingdon, UK). The mechanical properties of the sensor were evaluated by stretching and releasing it at a constant speed with an electronic tensile testing machine (ZQ-990B, Dongguan Zhitui Precision Instrument Co., Ltd., Dongguan, China). During the stretching tests, the resistance of the sensor was measured and recorded using a digital multimeter (Keysight 34465a, Keysight Technologies, Inc., Santa Rosa, CA, USA).

## 3. Results and Discussion

### 3.1. Microscopic Morphology and Structural Analysis

**Figure 2:**
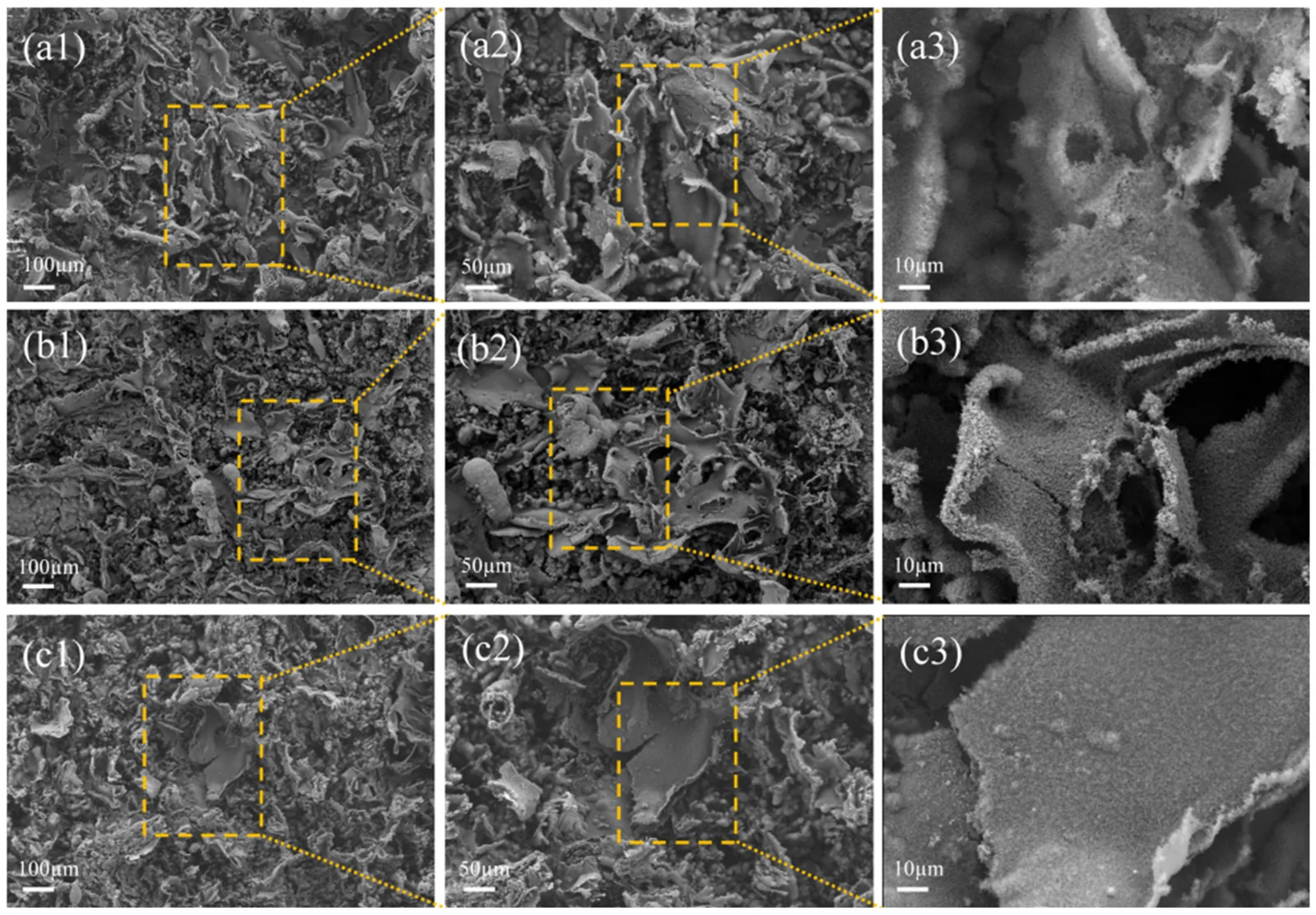
Scanning electron microscope (SEM) images of the three samples. (**a1**–**a3**) are the SEM images of Sample One, (**b1**–**b3**) are the SEM images of Sample Two, and (**c1**–**c3**) are the SEM images of Sample Three. The magnifications from left to right are 100 micrometers, 50 micrometers, and 10 micrometers, respectively.

The SEM images of the graphene sensors are shown in Figure 2. As can be observed, Sample 1 (Figure 2(a1–a3)) exhibits a relatively regular surface with a continuous film-like structure, featuring slight wrinkles and relatively large pores. Sample 2 (Figure 2(b1–b3)) displays a more complex surface, containing pores, cracks, and irregular structures, with some areas showing multilayer stacking or edge curling. Sample 3 (Figure 2(c1–c3)) presents an irregular but uniformly smooth surface with no obvious defects. These wrinkled structures are conducive to the formation of conductive pathways and can cause changes in resistance under stress, thereby achieving excellent sensing performance. Notably, as the proportion of PDMS increases, the surface pores of the samples gradually become smaller. The possible reason is that the reduced viscosity of the mixed system enhances material flowability and promotes more uniform dispersion of thermal decomposition products. Simultaneously, improved compatibility between PDMS and PI suppresses phase separation and contributes to the formation of a denser cross-linked network. These synergistic effects optimize the laser-induced carbonization process, reducing localized shrinkage, volatile gas accumulation, and interfacial defects. Consequently, the graphene structure becomes progressively denser, with reduced surface porosity and improved homogeneity.

**Figure 3:**
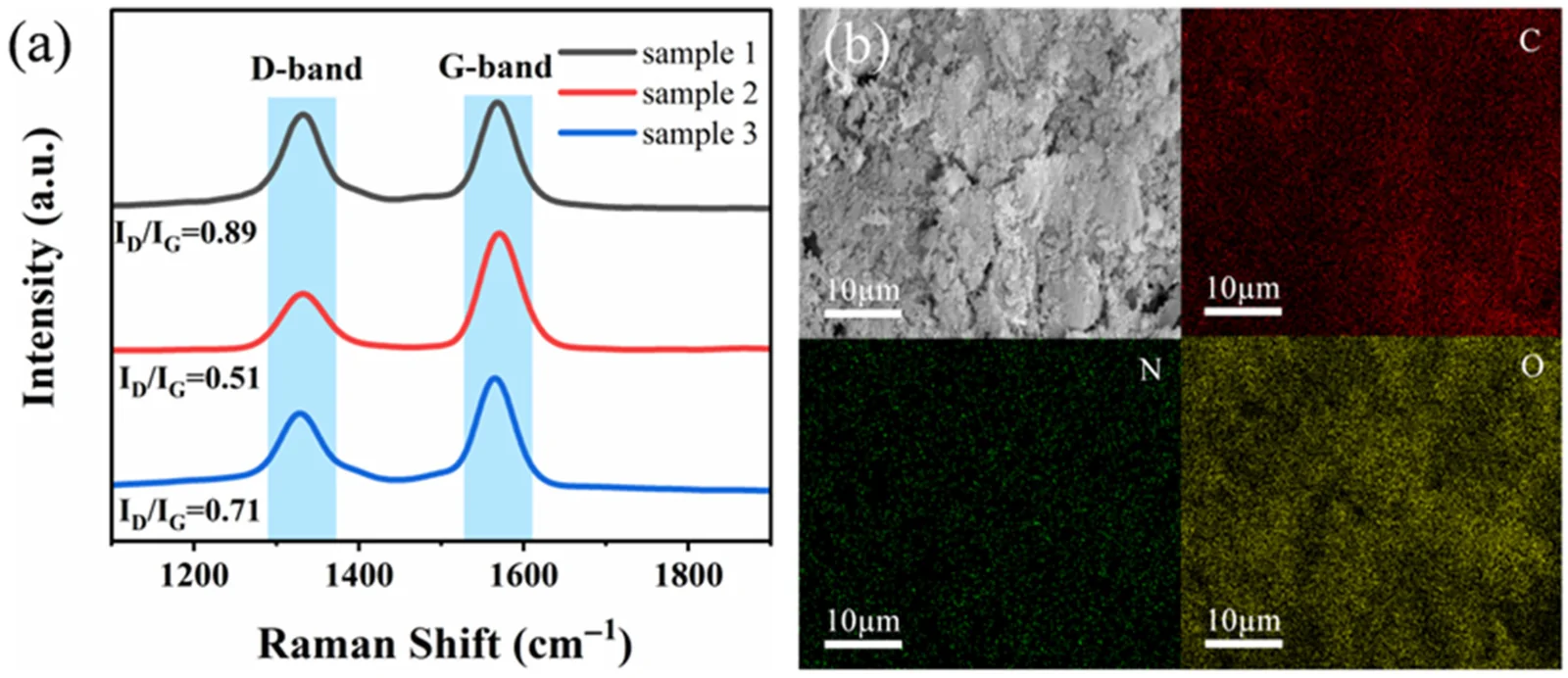
Raman spectrogram and EDS map. (**a**). Raman spectrograms of the three samples, from top to bottom they are Sample 1, Sample 2, and Sample 3. (**b**). Element mapping of Sample 2.

Typically, Raman scattering spectroscopy is employed to obtain information regarding the material’s structure, chemical composition, and physical properties. Figure 3a presents the Raman spectra of the three samples. All three samples exhibit two characteristic peaks at approximately 1350 cm^−1^ and 1580 cm^−1^, corresponding to the D (disordered or defective carbon) peak and the G (graphitic carbon) peak, respectively. The D peak represents the disorder-induced vibration mode in graphene and is commonly used to characterize structural defects. The G peak is the most prominent characteristic peak of graphene, arising from the interaction between C atoms. The presence and properties of graphene can be determined by measuring the position and intensity of the G peak. Meanwhile, the intensity ratio I_D_/I_G_ is used to evaluate the defect density or degree of disorder in the LIG. A higher I_D_/I_G_ ratio indicates a greater number of defects in the LIG sample. The I_D_/I_G_ values for Sample 1, Sample 2, and Sample 3 are 0.89, 0.51, and 0.71, respectively. This indicates that among the three samples, Sample 2 has the fewest defects and the most ordered structure, confirming that when the PDMS proportion increases to 1:3, the enhanced fluidity, compatibility, and crosslinking density effectively optimize the densification of graphene. However, the increase in the I_D_/I_G_ value of Sample 3 (PI:PDMS 1:4) to 0.71 suggests that an excessive amount of PDMS may lead to negative effects. Taking Sample 2 as an example, its energy-dispersive spectroscopy (EDS) mapping image is shown in Figure 3b, with the specific elemental contents listed in Table 1. It can be observed that C and O elements are uniformly distributed. The monomers of polyimide (PI) are typically diamines and dianhydrides, indicating that its structure should contain a significant amount of carbon, oxygen, and nitrogen. Because the laser-induced carbonization process converts the PDMS surface into a carbon-rich layer, the silicon signal from the underlying PDMS may be attenuated or obscured by this top carbonaceous layer. Separately, the absence of N element in the results can be explained by the fact that under laser irradiation, the C–O and C–N bonds in PI are broken, releasing O and N elements in gaseous form, while the C atoms recombine to form graphene. The presence of O is attributed to the originally high oxygen content in PDMS.

### 3.2. Sensing Performance Analysis

**Figure 4:**
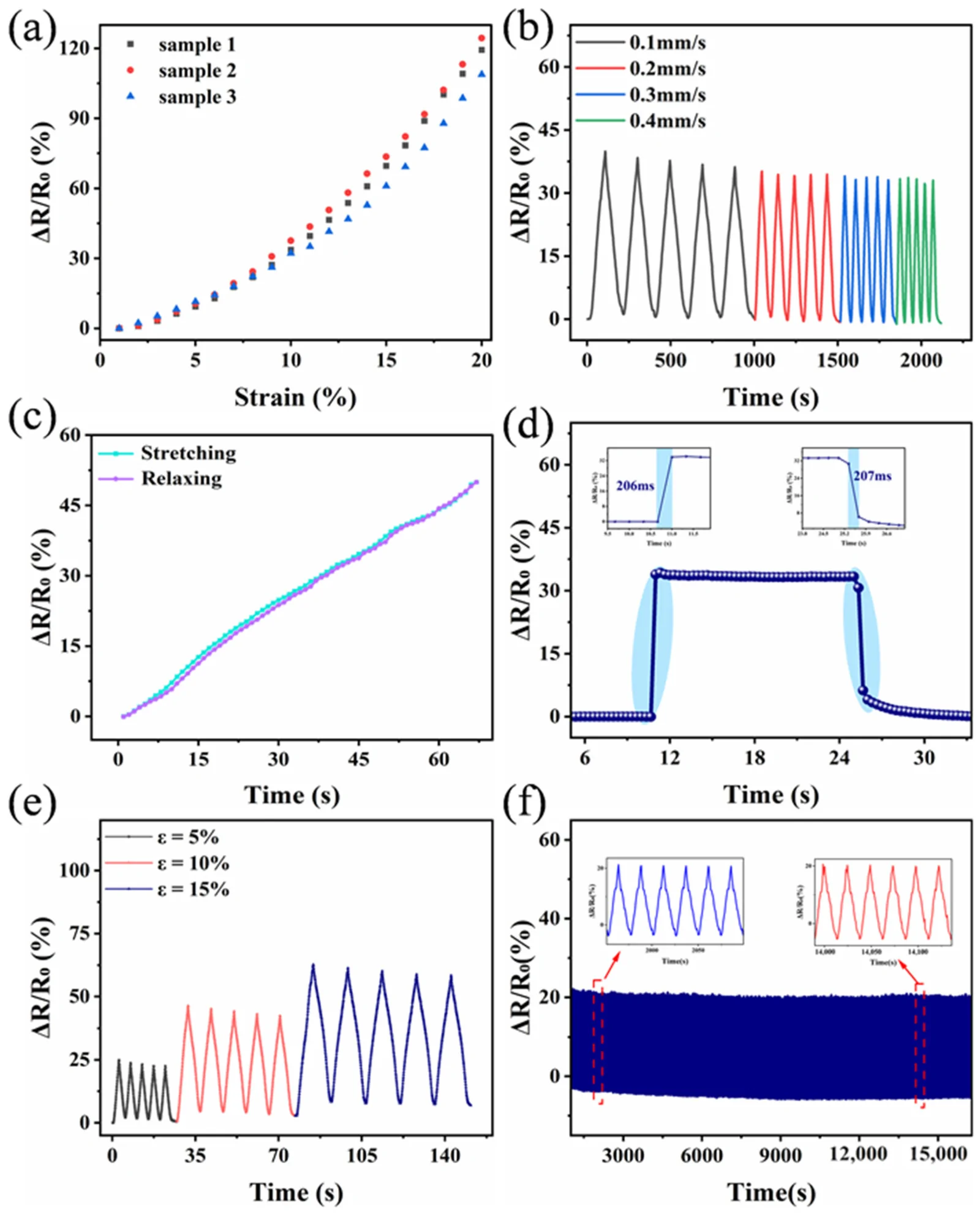
(**a**) Results of three samples under tensile stress within 20% strain range. (**b**) Changing the tensile speed while keeping the strain range at 10%. (**c**) Hysteresis curve. (**d**) Response time under loading and unloading conditions at 10% strain. (**e**) Keeping the strain range constant and changing it at a uniform speed. (**f**) Repeatability of the sensor. All the data are the results of single-device tests.

Linearity and sensitivity are the most fundamental and critical characteristics of a sensor. The initial assessment of whether a material can function as a sensor typically involves investigating its linearity and sensitivity. R^2^ (coefficient of determination) is a key metric used to evaluate the goodness of fit of a regression model and is commonly employed to characterize the linearity of data. Meanwhile, the sensitivity of a flexible strain sensor is often described by the relationship between strain and the change in resistance, quantified by the Gauge Factor (GF). The GF represents the relative change in resistance per unit strain and is typically used to characterize the sensor’s responsiveness to external strain, such as stretching or compression. Its calculation formula is GF = (Δ*R*/*R*_0_)/Δε, where Δ*R* is the resistance change, *R*_0_ is the initial resistance, and Δε is the applied strain. Specifically, it reflects the change in resistivity as a function of tensile deformation. Figure 4a shows the strain–resistance change curves of the three samples. It can be observed from the figure that the sensor exhibits piecewise linear characteristics: the first linear segment corresponds to a strain range of 0–10%, and the second segment corresponds to 10–20%. The underlying reason for this behavior may be tentatively attributed to the evolution of inter-sheet contacts within the graphene network. At low strain levels, the contact between graphene sheets changes minimally, resulting in a gradual and stable increase in resistance. As the strain increases, the contact between the sheets may begin to change more significantly, leading to an accelerated increase in resistance and forming a steeper linear segment. When the strain further increases, the resistance change plateaus or rises slowly. One plausible explanation is that some graphene sheet contacts break, and the resulting fragments may fill the pores between the sheets, re-establishing a stable conductive structure [28]. However, it should be noted that this sheet-fragment refilling mechanism remains a hypothesis at this stage, and alternative explanations cannot be excluded. For instance, the plateau could also arise from the saturation of contact-area reduction, the redistribution of conductive pathways, or the onset of tunneling-dominated conduction across separated sheets. Distinguishing among these possibilities requires further in situ characterization, such as real-time scanning electron microscopy or transmission electron microscopy under tensile loading combined with simultaneous resistance measurements.

To investigate the resistance variation characteristics of the sensor under different strain conditions, especially its performance under dynamic and long-term operation, performance analyses were conducted on all three samples. The following description takes Sample 2 as an example. Figure 4b shows the change in resistivity of the sensor when stretched at speeds of 0.1 mm/s, 0.2 mm/s, 0.3 mm/s, and 0.4 mm/s, respectively, while the strain range was maintained at 10%. The results indicate that the resistance change rates of the sensor are nearly identical under different stretching speeds, preliminarily demonstrating the stability of its sensing performance. Figure 4c displays the resistivity changes during stretching and returning within a 10% strain range. It can be observed that the two curves are closely aligned, indicating good hysteresis performance of the sensor. Figure 4d shows that under a 10% strain condition, the response time during stretching is 206 ms, and during returning is 207 ms. This not only reflects a short response time but also demonstrates that the sensor responds at a similar speed to opposite actions. Additionally, the magnified view in the upper part of the figure shows that the sensor performs stably during changes, without significant oscillations.

A comparison of the performance differences among the three samples is presented in Table 2, which includes linearity (R^2^), sensitivity (GF), response time, and the force required for failure. It should be noted that all the data are the results of single-sample tests. The R^2^ values are all above 0.96, with GF values of approximately 3.1 in the first stage and around 8.0 in the second stage. Among the three samples, Sample 2 exhibits the most superior performance, with the best linearity in the 10–20% strain segment, highest sensitivity, and shortest response time, among other advantages. This is consistent with its previously observed lowest I_D_/I_G_ value.

Figure 4e shows the rate of resistance change of the sensor under strain ranges of 5%, 10%, and 15%, respectively, at the same stretching and returning speed. It can be observed from the figure that the rate of resistance change is consistent for the same strain range and increases as the strain range expands. However, a slight increase in the rate of resistance change is noted at zero strain, which is likely due to contact or breakage of graphene flakes filling the gaps, leading to an increase in the resistance of the conductive pathways. Figure 4f illustrates the rate of resistance change of the sensor over 2000 repeated stretching cycles under the same strain and stretching speed. The thumbnail insets above, which are enlarged views of randomly selected sections on the left and right, demonstrate that the rate of resistance change is nearly identical in each stretching and returning cycle, further highlighting the stability of the sensor [29,30].

Overall, the reasons for achieving such excellent sensing performance can be summarized as follows. First, the graphene formed by laser-induced carbonization of the PI/PDMS blend possesses moderate conductivity, which is beneficial for resistance response at low strains. Second, the wrinkled structure and micropores formed during laser induction create a well-established conductive network that enhances both sensitivity and stability. Finally, the PI/PDMS blend is tightly bonded to the fabric, and the in situ-constructed LIG avoids interfacial detachment, thereby ensuring firm force transmission.

### 3.3. The Application of Flexible Strain Sensors

**Figure 5:**
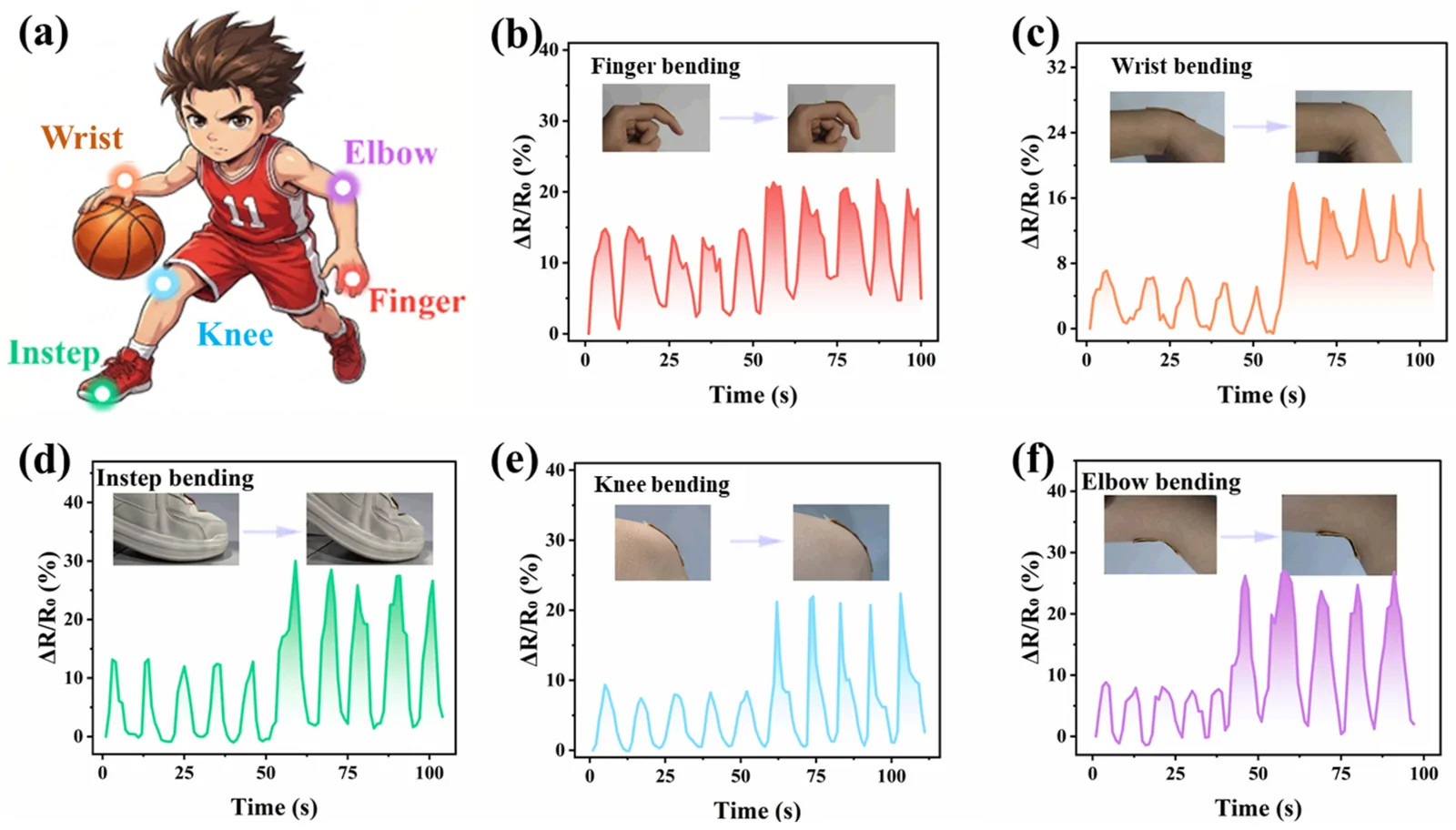
(**a**) Human body detection application. (**b**) Finger bending. (**c**) Wrist bending. (**d**) Instep bending. (**e**) Knee bending. (**f**) Elbow bending.

The graphene sensor developed in this study can serve as a wearable device for detecting physiological signals and human motion. Taking Sample 2 as an example, the sensor is capable of detecting physiological signals generated by different body joints, including finger bending, wrist bending, instep bending, knee bending, and elbow bending in Figure 5b–f, respectively. It should be noted that joint bending during physical activity can easily produce strains of 30–50%. In this study, the polymer precursor was directly coated onto the fabric surface and in situ-carbonized, so the fabric, rather than the sensing layer, contacts the skin. This configuration is expected to provide a mechanical buffering effect, in which the fabric substrate and attachment method distribute the deformation so that the strain experienced by the sensing element remains below 20% even under large joint bending. This buffering effect is an assumption based on the sensor configuration and was not measured directly in this work. As shown in Figure 5b, the flexible strain sensor was placed on the back of the finger, and the finger was bent to two different angles (Angle 1 and Angle 2) five times each, yielding the resistance change rate illustrated in the figure. Similarly, the flexible strain sensor was placed on the finger, wrist, instep, knee, and elbow, and each joint was bent to two angles five times. Comparing the same range of motion, the resistance change rates and variation curves were nearly identical, indicating stable performance of the sensor in practical applications. Comparing different ranges of motion, variations in resistance change were observed to be larger for some movements and smaller for others, reflecting that different body parts or bending degrees influence the sensor differently. This sensor can capture a variety of bending and twisting motions, making it suitable for applications in motion detection, health monitoring, and other fields. It can also be integrated into smart wearable devices to assist in detecting and analyzing human motion, or utilized in control systems for robotics and prosthetics [31,32].

On the other hand, the fabrication of the sensor involves impregnating the fabric with PI/PDMS at a specific ratio, followed by laser direct writing, which offers several distinct advantages over traditional preparation methods that do not incorporate fabric [33]. First, the fabric itself possesses excellent flexibility and comfort. When the sensor is integrated into the fabric, it maintains good flexibility and adaptability, making it more suitable for skin contact or wearing on various body parts. This enhances the wearing experience, particularly for applications such as motion monitoring or health tracking. Second, the fabric provides inherent resistance to stretching and bending, which effectively improves the mechanical stability of the sensor. The addition of the PI/PDMS composite not only ensures good flexibility but also maintains a certain degree of stretch resistance, thereby reducing the risk of damage or performance degradation during long-term use. Third, the fabric material allows for uniform coating of the PI/PDMS mixture through simple impregnation or coating processes. This method is compatible with existing textile manufacturing techniques, enabling large-scale production with cost advantages and ease of batch fabrication. Additionally, different direct-writing patterns can be designed according to specific requirements. Nevertheless, the washability and humidity sensitivity of the sensor have not been systematically evaluated in this work, which may affect its long-term reliability in practical wearable applications and should be addressed in future studies.

## 4. Conclusions

To address the issues of non-uniform dispersion leading to unstable sensing performance and poor uniformity in traditional graphene-based resistive sensors, which limit their applications, this study utilized a PI/PDMS precursor and laser-induced in situ formation of graphene on the fabric surface, significantly improving the stability of the graphene sensor performance. Simultaneously, the influence of the PI-to-PDMS ratio on laser-induced graphene was investigated. It was found that at a PI:PDMS ratio of 1:3, the formed graphene exhibited the fewest defects, the highest graphene content, and the most ordered structure, resulting in the best sensing performance. The sensor of sample 2 achieved a linearity of 0.99, a sensitivity within the 10–20% strain range of 8.64, and stable sensing performance over 2000 cycles. Additionally, when applied to human motion detection, the sensor demonstrated favorable stability. In conclusion, this study provides a method for the rapid, stable, and in situ fabrication of graphene resistive sensors on fabric surfaces, showcasing promising application prospects in terms of skin-friendly contact and high stability.

## Figures and Tables

**Table 1:** Total Spectrum of Distribution Graphs (The EDS results reflect the composition of the carbonized surface layer).

Element	Characterization Method	Wt%	At%
C	EDS	42.91	50.03
N	EDS	0.00	0.00
O	EDS	57.09	49.97
Total		100.00	100.00

**Table 2:** Comparison of Sensing Performance Differences among Three Samples.

Sample	R^2^ (0–10%)	GF (0–10%)	R^2^ (10–20%)	GF (10–20%)	Loading Response Time (ms)	Recovery Response Time (ms)	Breaking Force (N)
1	0.97	3.14	0.99	8.38	207	207	23.6
2	0.97	3.48	0.99	8.64	206	207	20.4
3	0.99	3.12	0.97	7.63	210	211	22.6

## Data Availability

The original contributions presented in the study are included in the article. Further inquiries can be directed to the corresponding author.
